# Lens development depends on a pair of highly conserved *Sox21* regulatory elements

**DOI:** 10.1016/j.ydbio.2012.02.025

**Published:** 2012-05-01

**Authors:** Stefan Pauls, Sarah F. Smith, Greg Elgar

**Affiliations:** Division of Systems Biology, MRC National Institute for Medical Research, The Ridgeway, Mill Hill, London NW7 1AA, UK

**Keywords:** Sox21, Conserved non-coding elements, Lens, Zebrafish, Enhancer

## Abstract

Highly conserved non-coding elements (CNEs) linked to genes involved in embryonic development have been hypothesised to correspond to cis-regulatory modules due to their ability to induce tissue-specific expression patterns. However, attempts to prove their requirement for normal development or for the correct expression of the genes they are associated with have yielded conflicting results. Here, we show that CNEs at the vertebrate *Sox21* locus are crucial for *Sox21* expression in the embryonic lens and that loss of Sox21 function interferes with normal lens development. Using different expression assays in zebrafish we find that two CNEs linked to *Sox21* in all vertebrates contain lens enhancers and that their removal from a reporter BAC abolishes lens expression. Furthermore inhibition of Sox21 function after the injection of a *sox21b* morpholino into zebrafish leads to defects in lens development. These findings identify a direct link between sequence conservation and genomic function of regulatory sequences. In addition to this we provide evidence that putative Sox binding sites in one of the CNEs are essential for induction of lens expression as well as enhancer function in the CNS. Our results show that CNEs identified in pufferfish-mammal whole-genome comparisons are crucial developmental enhancers and hence essential components of gene regulatory networks underlying vertebrate embryogenesis.

## Highlights

► Conserved non-coding elements (CNEs) linked to Sox21 contain two lens enhancers. ► Removal of these enhancers from a reporter BAC abolishes lens expression. ► Morpholino knockdown of zebrafish sox21b leads to defects in lens development. ► Conserved putative Sox binding sites are vital for enhancer activity in lens and CNS. ► CNEs are crucial for normal development and contain overlapping embryonic enhancers.

## Introduction

Cis-regulatory modules (CRMs) are crucial determinants of tissue-specific expression patterns during development. They can be described as distinct sequence blocks of a few hundred nucleotides that contain multiple binding sites for transcription factors (Howard and Davidson, 2004). Since sequence signatures that generally define CRMs are still unknown evolutionary conservation of non-coding sequences has been used in a number of whole genome approaches to identify CRMs in vertebrates (Bejerano et al., 2004; Couronne et al., 2003; McEwen et al., 2009; Pennacchio et al., 2006; Sandelin et al., 2004b; Venkatesh et al., 2006; Woolfe et al., 2005). However, attempts to address the significance of these so-called conserved non-coding elements (CNEs) for regulating a specific gene locus have yielded conflicting results and consequently this still remains an important but largely unanswered question.

Sequence conservation of non-coding DNA at isolated gene loci is routinely used to identify putative regulatory sequences. When high quality genome data became available it was possible to expand the search for CNEs to entire genomes. It was found that CNEs tend to be clustered around developmental genes (Bejerano et al., 2004; McEwen et al., 2009; Pennacchio et al., 2006; Sandelin et al., 2004b; Woolfe et al., 2005) and that many of them induce tissue-specific reporter gene expression in early embryos (McEwen et al., 2009; Pennacchio et al., 2006; Woolfe et al., 2005) suggesting that they represent a set of regulatory sequences indispensable for vertebrate development. Furthermore, it was found that single nucleotide changes occurring in CNEs can be linked to severe diseases such as holoprosencephaly (Jeong et al., 2008) and autism (Poitras et al., 2010). However, there are surprisingly few examples where it was possible to establish a direct link between CNEs and embryonic development by deleting them from their genomic locus. A notable exception was the removal of two conserved non-coding sequences from the *Sonic hedgehog* locus causing the degeneration of skeletal elements in the limb in one case (Sagai et al., 2005) and hypoplasia of a number of tissues normally derived from the pharynx in the other (Sagai et al., 2009). In contrast to these findings other loss-of-function studies failed to deliver the proof that these sequences are crucial for normal development, as suggested by the fact that they have remained almost unchanged after 450 Million years of evolution. Two large-scale deletions induced in the mouse genome each removing several hundreds of non-coding regions conserved between rodents and humans resulted in viable mice although both deletions affected gene expression in one neighbouring gene (Nobrega et al., 2004). In a second report four CNEs from different gene loci were deleted separately but knock-out mice neither developed any obvious abnormal phenotypes nor revealed any changes in gene expression (Ahituv et al., 2007). It is therefore still unclear at the moment whether sequence conservation alone is a reliable indicator for detecting regulatory sequences in vertebrate genomes.

Here we establish a functional link between CNEs present at the *Sox21* loci of all jawed vertebrates and lens development in zebrafish. *Sox21* is expressed in the developing lens in chicken and zebrafish (Lan et al., 2011; Uchikawa et al., 1999) but its function for lens development is currently unknown. Using transient transgenesis in zebrafish we conducted a complete survey of CNEs at the Fugu *Sox21* locus and identified two that were active in the developing lens. Furthermore, we use BAC recombineering and morpholino knock-down of zebrafish Sox21b to show that these CNEs are crucial for *Sox21* induction in the lens and that *sox21b* is crucial for lens development. Finally, we dissect one of the lens enhancers in greater detail and find an overlap of enhancer functions in the lens and the CNS with implications for the evolution of these highly conserved regulatory sequences.

## Results

### Lens enhancers linked to *Sox21* are situated in CNEs

**Fig. 1:**
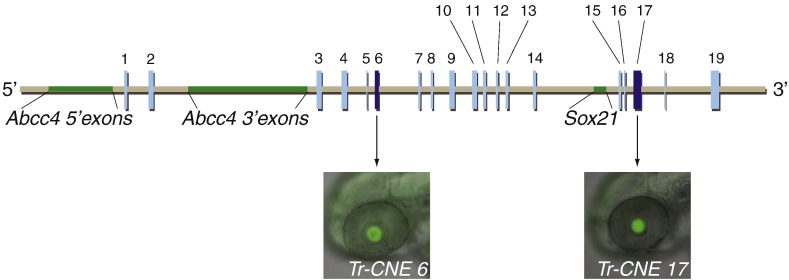
*Sox21* CNEs containing lens enhancers. Schematic representation of the vertebrate *Sox21* locus with coding regions in green and CNEs in blue. Two CNEs (1 and 2) are situated in an intron of the multi-exon *Abcc4* gene upstream of *Sox21* in all vertebrates. The co-injection assay indentified two Fugu CNEs (6 and 17), in dark blue, that upregulate GFP expression in the lens of 52 hpf old zebrafish. Tr (*T**akifugu**r**ubripes*, Fugu).

First we wanted to establish the tissue-specificities for all CNEs associated with one locus and then select a subset with overlapping activities for further analysis. We chose the *Sox21* locus for this purpose due to its limited number of CNEs and also because, in addition to the CNS, it is expressed in a number of sensory organs (Cunningham, et al., 2008; Lan et al., 2011; Rex et al., 1997; Rimini et al., 1999; Uchikawa et al., 1999) which in zebrafish are easily accessible for analysing reporter gene expression. In fact some of the *Sox21* CNEs when tested before drove expression in the CNS but also in the developing eye and ear (Woolfe et al., 2005). All *Sox21* CNEs can be retrieved from the CONDOR database (Woolfe et al., 2007) which has 31 entries for this locus. Conservation peaks close to each other suggest that some of the CNEs defined in silico most probably form a functional unit in vivo. Therefore we clustered some of the *Sox21* CNEs resulting in 19 distinct CNEs for further analysis (Fig. 1, Table S1).

**Fig. 2:**
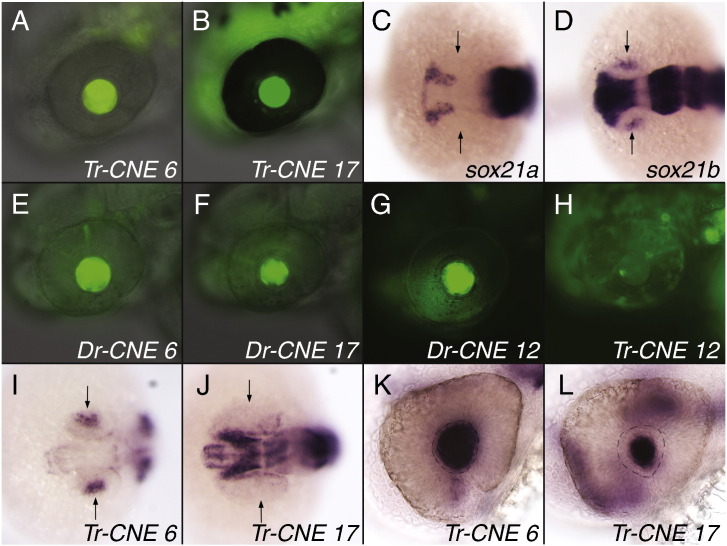
*GFP* and *Sox21* expression in the zebrafish lens. (A, B) Stable transgenic lines expressing *GFP* in the lens under the control of Fugu CNE 6 (A) and Fugu CNE 17 (B) at 52 hpf. (C, D) *sox21a* and *sox21b* expression in zebrafish at the 20 somite stage. (E–H) Transient *GFP*-expression in the lens at 52 hpf activated by zebrafish CNE 6 (E), zebrafish CNE 17 (F) and zebrafish CNE 12 (G). In contrast to the zebrafish orthologue Fugu CNE 12 is not active in the lens (H). (I–L) *GFP* expression in stable transgenic lines. Only CNE 6 is active in the lens at 20 somites (I) whereas CNE 17 activity at this stage is limited to the CNS (J). At 30 hpf also CNE 17 is active in the lens (L) but in a more central domain than CNE 6 which seems to drive expression throughout the lens (K). (A, B, E–H) *GFP* fluorescence, anterior to the left. (C, D, I–L) Whole-mount in situ hybridizations, anterior to the left. Dorsal view (C, D, I, J), lateral view (K, L). Arrows indicate position of the lens during early stages. A dashed line marks the boundary of the lens in K and L. Tr (*T**akifugu**r**ubripes*, Fugu), Dr (*Danio rerio*, zebrafish).

**Fig. 3:**
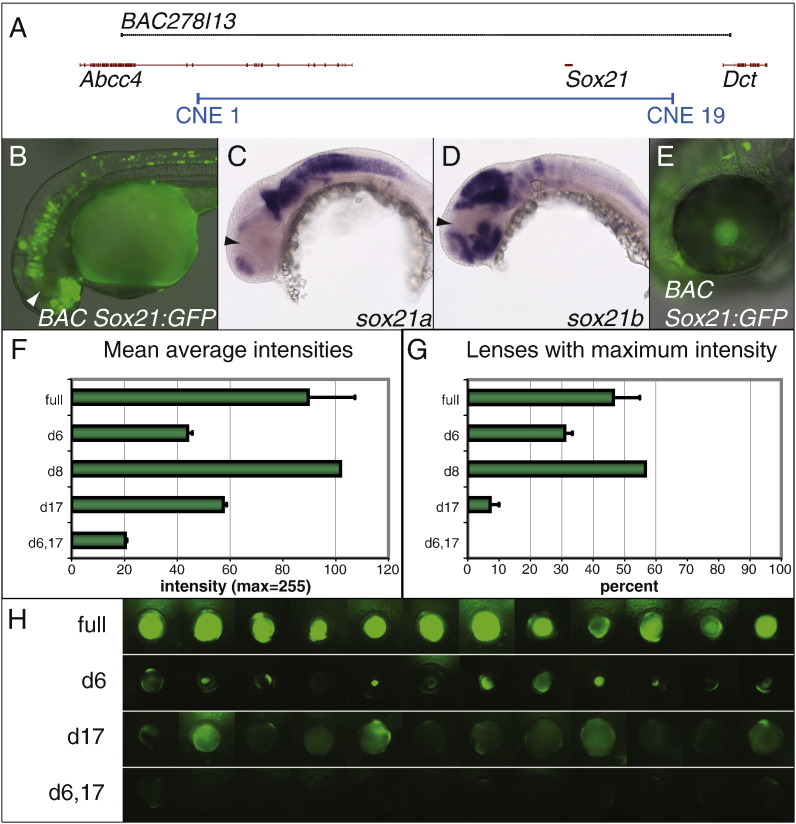
Lens expression from a Fugu *Sox21* BAC. (A) Genomic region included in Fugu BAC278I13. The BAC is shown as a black line, exons in red and the region including the *Sox21* CNEs in blue. The CNE region is shown by indicating the first upstream CNE and the last downstream CNE. (B) Transient expression of BAC *Sox21:GFP* in zebrafish at 30 hpf. (C, D) Whole-mount in situ hybridizations for zebrafish *sox21a* (C) and *sox21b* (D) at 30 hpf. (B–D) Absence of expression in the dorsal diencephalon is indicated with arrowheads. (E) Transient expression of BAC *Sox21:GFP* in the zebrafish lens at 52 hpf. (F, G) Intensity of *GFP*-fluorescence in lenses after the injection of different variants of BAC *Sox21:GFP*. Error bars indicate standard error of the mean. (F) Mean average intensities of lens expression. (G) Percentage of lenses with maximum levels of intensity. full = BAC *Sox21:GFP* including all CNEs (*n* = 51); d6 = deletion of CNE 6 (*n* = 51); d8 = deletion of CNE 8 (*n* = 30); d17 = deletion of CNE 17 (*n* = 43); d6,17 = double deletion CNE 6/CNE 17 (*n* = 25). (H) Lens expression of different variants of BAC *Sox21:GFP*. Each row shows 12 representative images of transient *GFP* expression in the lens at 52 hpf. Note that *GFP* expression in row ‘d6’ tends to be restricted to the centre of the lens whereas residual *GFP*-expression in row ‘d17’ is often detected in the entire lens. Moreover, the construct lacking both CNEs (d6, 17) performs consistently worse than the single deletions and fluorescence never exceeds background levels. Abbreviations of BAC constructs as in (F, G).

To determine the tissue-specificity of the entire *Sox21* CNE complement we applied the same co-injection assay as previously (Muller et al., 1997; Woolfe et al., 2005) using Fugu sequences. Most of the *Sox21* CNEs are able to generate tissue-specific expression patterns in the CNS and sensory organs (Tables S2, S3) in good agreement with *Sox21* expression in the same tissues (Cunningham, et al., 2008; Lan et al., 2011; Rex et al., 1997; Rimini et al., 1999; Uchikawa et al., 1999; Figs. 3B, C). Concentrating on sensory organs there is a number of CNEs harbouring otic enhancers but only two CNEs specific for the lens, CNEs 6 and 17 (Fig. 1) which we selected for further analysis. To verify the results from the co-injection assay in a second independent assay we generated stable transgenic zebrafish lines using the tol2 system (Kawakami et al., 2004). This confirmed the presence of lens enhancers in CNE 6 and 17 (Fig. 2A, B) as well as the accuracy of the co-injection assay.

Zebrafish have two copies of *Sox21*, called *sox21a* and *sox21b*. Only *sox21b* is expressed in the lens (Lan et al., 2011) and *sox21b* expression starts at 20 somites (Fig. 2C, D). Consequently we find sequences corresponding to CNE 6 and CNE 17 only at the *sox21b* locus. Given the degree of sequence similarity with the orthologous Fugu sequences we expected to find lens enhancers in the zebrafish sequences as well. In fact, when tested in the tol2-system the zebrafish CNEs are also able to upregulate *GFP* in the lens (Fig. 2E, F). However, when testing additional zebrafish sequences we found a third lens enhancer in the CNE 12 orthologue at the *sox21b* locus (Fig. 2G) which again is absent from the *sox21a* locus. This suggests differences in the regulatory architecture guiding lens expression in zebrafish and Fugu since neither the co-injection assay (Tables S2,S3) nor a tol2-based analysis (Fig. 2H) found evidence for a lens enhancer in Fugu CNE 12.

An explanation for the presence of two separate highly conserved lens enhancers associated with *Sox21* could be that they are functionally distinct. Therefore we determined the time-course of *GFP* expression in transgenic lines for both CNE 6 and CNE 17. We found that whereas Fugu CNE 6 activates lens expression as early as the 20-somite stage (Fig. 2I), the same stage when zebrafish *sox21b* starts being expressed in the lens (Fig. 2D), Fugu CNE 17 is only active much later at 30 hpf (Fig. 2J, L). We also noted spatial differences in the distribution of *GFP* mRNA in the transgenics. The Fugu CNE 6 lines express *GFP* throughout the lens whereas Fugu CNE 17 activity seems to be restricted to the central part of the lens (Fig. 2K, L). From this we conclude that both lens enhancers receive different regulatory input and regulate different aspects of *Sox21* lens expression.

### Removal of the conserved lens enhancers from the *Sox21* locus abolishes lens expression

Next we addressed the question whether *Sox21* lens expression depends on the CNE 6 and 17 lens enhancers and whether there were other non-conserved lens enhancers present at the same locus. Therefore we tested the response of the entire *Sox21* locus to the removal of both CNEs using a Fugu *Sox21* BAC, including all 19 CNEs (Fig. 3A), in which the single *Sox21* exon was replaced with a *GFP* coding sequence. We chose to analyse the Fugu *Sox21* locus because it is much more compact than the zebrafish *sox21b* locus. Moreover, the *Sox21* gene duplication that seems to be unique to zebrafish suggests that the Fugu locus and its CNEs are more representative for other vertebrate Sox21 loci.

Transient expression from the BAC in zebrafish was in good agreement with *Sox21* expression in the CNS and more importantly the eye (Fig. 3B–E). Most regions of the CNS express at least one of the two zebrafish *Sox21* genes at 30 hpf with the exception of the dorsal diencephalon (Fig. 3C, D). *GFP* expression from the BAC can also be detected throughout the entire CNS but not in the dorsal diencephalon (Fig. 3B). Next we removed CNE 6 and 17 from the BAC and assayed the consequence of this loss for *GFP* expression in the lens (Fig. 3F–H). This was done by measuring the mean average intensity of *GFP* fluorescence in the lenses of zebrafish embryos injected with the modified BACs (Fig. 3F). We found that removal of either CNE 6 or CNE 17 but not CNE 8, a CNE with weak lens activity in the co-injection assay, leads to a significant drop in the intensities compared to the entire locus. This effect is further increased in the CNE 6/CNE 17 double deletion where fluorescence hardly exceeds background levels (Fig. 3H). This suggests that both enhancers are required to reach full levels of gene expression and that they act independently from each other. Similarly, when counting the number of lenses showing maximum intensities of *GFP* expression (Fig. 3G) we also found that removing both CNEs had the strongest effect resulting in a complete absence of high levels of gene expression. Loss of each of the CNEs on its own did affect maximum levels as well with CNE 17 showing a stronger effect than CNE 6 whereas deletion of CNE 8 did not affect maximum intensities. Moreover, we noticed that after the loss of CNE 6 residual *GFP* expression tends to be localized at the centre of the lens whereas it is uniform but weak after the loss of CNE 17 (Fig. 3H). These observations accurately reflect the different spatial activities of both lens enhancers (Fig. 2K, L). In summary this suggests that both CNEs are required for *Sox21* lens expression and that no other CNE, including CNE 12, or non-conserved sequence at the Fugu locus can compensate for the loss of the CNE 6 and CNE 17 enhancers.

### Zebrafish Sox21b is crucial for lens development

**Fig. 4:**
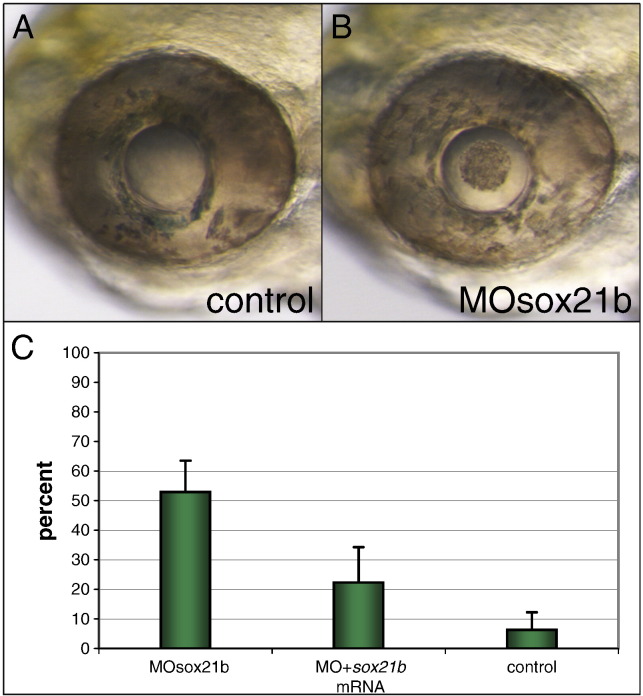
Morpholino knock-down of zebrafish Sox21b. (A) Lens of a control embryo injected with a 5 bp-mismatch morpholino at 3 dpf. (B) Lens of a morpholino-injected embryo at 3 dpf. (C) Percentage of embryos showing the same lens phenotype as in (B) when injected with the morpholino (MOsox21b), the morpholino together with the *sox21b* mRNA (MO + *sox21b* mRNA) or with the 5 bp-mismatch morpholino (control). Error bars indicate standard error of the mean.

An important question that emerges from the BAC data is whether loss of *Sox21* expression in the lens would also interfere with normal lens development. The extraordinarily high degree of sequence conservation of the two lens enhancers suggests that they are crucial during this process. Therefore we decided to address the loss of Sox21 function on lens development by injecting a *sox21b* morpholino into zebrafish. This led to a pronounced morphological defect in the lens of over half of the injected embryos at 72 hpf (Fig. 4). Differing from control embryos, the morphants developed a patch of aberrant tissue in the centre of the lens indicative of defects during lens fibre differentiation (Fig. 4A, B). We confirmed specificity of the morpholino by using a mismatch morpholino that resulted in only very few defective lenses and we were able to partially rescue the phenotype by injecting *sox21b* mRNA (Fig. 4C). Taken together these findings strongly suggest that absence of *Sox21* expression in the lens due to the loss of the CNE 6 and CNE 17 lens enhancers would also disrupt lens development and may explain the presence of these sequences in all vertebrate genomes.

### Dissection of CNE6

**Fig. 5:**
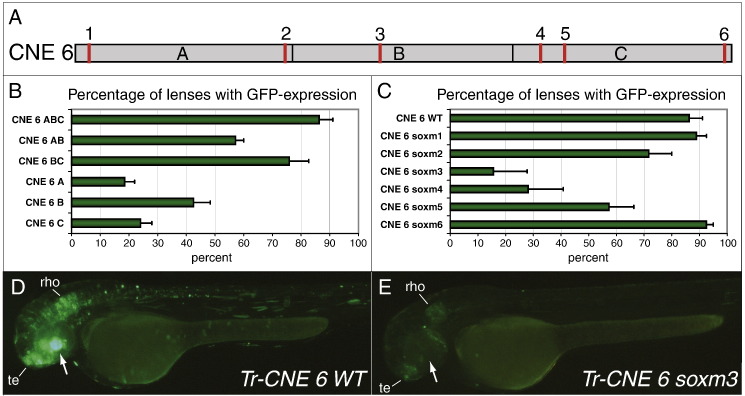
Dissection of the Fugu CNE 6 lens enhancer. (A) Schematic drawing of CNE 6 to indicate the subdivision into three parts (ABC) for the deletion analysis and the position of the six putative Sox binding sites (red) numbered 1–6. (B) Percentage of *GFP*-positive lenses in zebrafish injected with various deletion clones of Fugu CNE 6 (ABC = full-length CNE 6). (C) Percentage of *GFP*-positive lenses in zebrafish injected with different mutated versions of CNE 6. All 6 putative Sox sites were mutated separately (soxm1-6). (D,E) Transient *GFP* expression in 52 hpf-old zebrafish injected with the Fugu CNE 6 WT sequence (D) or a variant carrying a mutation in putative Sox site 3 (E). The lens is indicated by an arrow. Note that absence of lens expression in (E) correlates with lower expression levels in telencephalon (te) and rhombencephalon (rho). Tr (Takifugu rubripes, Fugu).

Finally we analysed the early lens enhancer in CNE 6 in more detail to address two questions regarding the organisation of this enhancer in general. First, we asked whether the need to conserve the lens enhancer could explain the presence of CNE 6 in all vertebrates. If this was the case a considerable part of the conserved sequence should be essential for lens expression and not just one or two isolated binding sites. Second, CNE 6 is also active in the CNS and this raises the question whether both functions overlap and the same sequences or even binding sites are used by different tissue-specific regulatory circuits. We started by dissecting the Fugu CNE into three regions of approximately equal size (Figs. 5A and S1A) and generated tol2 reporter constructs to examine whether any of them was dispensable for lens expression. After injecting these constructs into zebrafish we counted the number of *GFP*-positive lenses in the injected embryos and found that only the entire CNE was able to induce full levels of *GFP* expression in the lens (Fig. 5B). Amongst the three sub-regions, the central part of the CNE was a slightly stronger activator than the flanking regions and in combination with the 3' part it reaches almost WT levels. This suggests the presence of some crucial transcription factor binding sites in that area and that the CNE 6 lens enhancer is rather complex requiring most of the conserved sequence.

It has been shown that the SoxB1 genes *Sox1*, *Sox2* and *Sox3* are involved in lens development and *Crystallin* gene expression in mouse and chick (Kamachi et al., 1995, 1998; Nishiguchi et al., 1998) and in the nervous system the balanced activity of SoxB1 and SoxB2 genes, such as *Sox21*, is crucial for proper development of neurons from undifferentiated precursors (Sandberg et al., 2005). Therefore we scanned Fugu CNE 6 for the presence of putative Sox binding sites and considered 6 evolutionary conserved putative sites for further analysis (Figs. 5A, S1A). Four of these sites are conserved between Fugu, zebrafish, Xenopus, chick, mouse and human, one between Fugu, chick, mouse and human whilst one seems to be conserved only in fish. We injected tol2 clones harbouring mutations for each single site and, as before, counted the number of *GFP*-positive lenses in the injected embryos (Fig. 5C). We found that whereas sites 1 and 6 are dispensable for lens expression, absence of sites 2 and 5 reduces, and loss of sites 3 or 4 almost entirely abolishes, enhancer activity. The fact that the removal of the only site situated in the central part of CNE 6 also results in the steepest decline in lens activity is in good agreement with our observation that the central part on its own is a more potent activator than the two flanks. Similarly, combination of sites 3 and 4 in the BC deletion construct may explain why the 5' part of CNE 6 seems to be the most dispensable region for lens expression.

This analysis also revealed that there is overlap between the lens and the CNS enhancer in CNE 6. Whereas the WT CNE is also strongly active in telencephalon and rhombencephalon (Fig. 5D) a construct carrying a mutation in site 3 is a far less potent activator in these brain areas (Fig. 5E). Expression in the brain was also affected after mutating site 4 (Fig. S1B, C) which suggests that lens and CNS enhancer either depend on the same binding sites or distinct but overlapping binding sites. This demonstrates that the lens enhancer in CNE 6 depends on a minimum of four distinct binding sites spanning a region of 179 bp and that two of them are equally important for expression in lens and CNS.

## Discussion

Evolutionary conservation of non-coding sequences is widely used to identify CRMs despite the fact that the genomic function of the vast majority of these conserved sequences remains unknown. Our analysis of *Sox21* regulation in the lens shows that it depends on two functionally distinct enhancers situated in highly conserved sequences present in the genomes of all jawed vertebrates. The degree of sequence conservation of these enhancers reflects their significance for development because their removal leads to the absence of lens expression at a locus crucial for lens development. This is one of the few examples in which targeting of highly conserved sequences derived from whole-genome comparisons has revealed a crucial regulatory function during development.

CNE datasets vary between each other depending on the species and conservation thresholds used for the whole-genome comparisons. Nevertheless, it is well established that highly conserved non-coding sequences in vertebrates tend to be clustered around genes involved in embryonic development. However, at least some of these sequences seem to be dispensable since their removal from the genome does not perturb normal development (Ahituv et al., 2007; Nobrega et al., 2004). One explanation for these negative findings might be the robustness of developmental gene expression. This is an important aspect because embryonic development crucially depends on the precise establishment of a number of highly dynamic and complex gene expression patterns. Some of these genetic loci have several distinct enhancers that generate very similar expression patterns when tested in isolation in so-called gain of function assays. Moreover it has been shown that in some cases reporter gene expression in a specific tissue is only affected after the loss of multiple distinct enhancers from a single locus (Jeong et al., 2006). The presence of these seemingly redundant enhancers may be explained by the fact that they increase robustness by complementing each other. However, this also means that such a pair of enhancers would be under weaker evolutionary constraints and therefore with respect to CNEs the question is whether a regulatory sequence can be both redundant and highly conserved. Usually evidence for the redundancy of regulatory sequences is gathered in a controlled lab environment but recent results show that the same sequences may lose the ability to complement each other if these conditions change. In Drosophila loss of seemingly redundant enhancers can be tolerated if development proceeds under optimal environmental and genetic conditions but not if these parameters change (Frankel et al., 2010; Perry et al., 2010). In the wild where developmental gene expression patterns have to be established under a number of very different conditions the robustness of these patterns is a crucial factor for survival which is thus a target for natural selection. This means that regulatory sequences like CNEs are not only under purifying selection for their role in transcriptional regulation but also to guarantee the robustness of these transcription patterns. This may also explain the negative findings in the aforementioned studies (Ahituv et al., 2007; Nobrega et al., 2004). Even though the removal of CNEs from the genome of highly inbred lab animals does not lead to any obvious defects this may not be the case when moving to a different genetic background or after changing external parameters such as temperature or diet.

Robustness of lens expression, however, is probably not the reason for the presence of two distinct lens enhancers at the *Sox21* locus. Given that before 30 hpf *Sox21* lens expression is exclusively regulated by CNE 6 and that later both enhancers show different spatial activities it seems unlikely that they can fully complement each other. Moreover the fact that the early enhancer is active also in more peripheral regions of the lens suggests that it might be active in lens fibre precursors situated in the lens epithelium. On the other hand the late enhancer in CNE 17 is not active in peripheral lens cells but probably only in differentiated lens fibres in the centre of the lens that first appear in zebrafish at 36 hpf (Dahm et al., 2007) which is shortly after the onset of gene expression from this enhancer. Differences in the regulatory networks of lens precursors and differentiated lens fibres may also account for the presence of two distinct enhancers. One such difference could involve a SoxB1–SoxB2 interplay present in precursors to trigger differentiation but absent in differentiated lens fibre cells. Since targeting of Sox consensus binding sites in CNE 6 can abolish lens expression from the early *Sox21* lens enhancer an interplay between SoxB1 activator and SoxB2 repressor proteins, as described for the nervous system (Sandberg et al., 2005), could also be involved during lens expression. Similar to the CNS it is possible that *SoxB1* genes are needed to maintain the precursor pool in the lens epithelium and that their repression by Sox21 triggers differentiation. Subsequently however, SoxB1 expression has to be restored because it is crucial for *Crystallin* gene expression in lens fibre cells of chicken and mice (Kamachi et al., 1995, 1998; Nishiguchi et al., 1998). However, these cells also express Sox21 and this means that whereas *Sox2* repression by Sox21 may be an essential part of the regulatory network in lens fibre precursors *Sox2* has to be unresponsive to Sox21 in lens fibre cells. This could be achieved by two distinct enhancers at the *Sox2* locus of which only the early one but not the late one would be repressed by Sox21. In fact a separation of early and late lens expression has been described not only for *Sox21* but also for the *Sox2* regulatory region (Uchikawa et al., 2003) although it is currently unknown whether any of the two enhancers is directly regulated by Sox21. Similarly, SoxB1 genes although necessary are probably not sufficient to activate gene expression from the early *Sox21* lens enhancer. If SoxB1 gene expression on its own was sufficient to induce *Sox21* this would lead to a rapid depletion of the precursor pool because of the inhibitory effect of Sox21 on SoxB1 genes which are needed for precursor pool maintenance. Instead, it is more likely that other factors in addition to *SoxB1* genes are required to guarantee that Sox21 initiates differentiation not in all but just in a subset of precursors. These factors, however, may not continue to be expressed in differentiating lens fibres where in theory *SoxB1* gene expression would be sufficient to induce Sox21 expression because Sox21 does not induce negative feedback on SoxB1 genes in these cells. Therefore different regulatory states in precursors and differentiating lens fibres, as illustrated by a possible SoxB1–SoxB2 interaction during lens fibre differentiation, may account for the presence of two distinct *Sox21* lens enhancers.

The fact that all regulatory sequences responsible for *Sox21* expression in the lens reside in CNEs suggests that *Sox21* is involved in lens development in all vertebrates. In fact, CNE 17 may perhaps be considered the most conserved enhancer in all metazoans because a regulatory sequence corresponding to its 3' region was recently identified even in Cnidaria (Royo et al., 2011). However, data on *Sox21* lens expression in species other than zebrafish and chick are not available at this moment and no lens phenotype was mentioned in a recently published *Sox21* mouse knock-out (Kiso et al., 2009). Therefore it is an important question whether alternative mechanisms exist that would explain the conservation of the two lens enhancers independently of Sox21 function for lens development. Indeed another explanation for the retention of the enhancers could be their overlap with other tissue-specific enhancers as seen in CNE 6. Such an overlap of enhancer functions in highly conserved regulatory sequences has recently been shown also for a CRM associated with *Sox10* (Betancur et al., 2011). This *Sox10* regulatory sequence is active in neural crest and otic placode and it was shown that a triplet of different but paralogous transcription factors uses the same binding sites to activate gene expression in these two tissues. This means that purifying selection acting on the CNS enhancer would also lead to the conservation of the lens enhancer even if it were dispensable. The possibility that Sox21 function during lens development is not required in species other than zebrafish may also be illustrated by the presence of a third lens enhancer in zebrafish CNE12 which is absent at least in Fugu. On the other hand it is striking that this additional enhancer is situated in a CNE and the modification of highly conserved regulatory sequences may be problematic in species that did not undergo a duplication of the *Sox21* locus. Zebrafish appears to be the only species with a duplicated *Sox21* locus and as a consequence the only one where this locus has experienced a period of relaxed evolutionary constraint. If this were a prerequisite to modify highly conserved enhancers it would explain the absence of a third CNE with lens activity in other species. The function of the third lens enhancer in zebrafish is unknown so far but may again help to increase robustness of Sox21 lens expression which of course raises the question why this would not be necessary in other species if they were also dependent on Sox21 function for lens development. As a matter of fact this may be achieved differently in species with a single *Sox21* locus by for instance expressing other genes in the lens that can compensate for fluctuations in Sox21 expression. This could also explain the absence of a lens phenotype in the *Sox21* mouse knock-out (Kiso et al., 2009). In such a scenario the overlap of lens enhancers and other tissue-specific enhancers would gain importance as well because if a second locus could complement *Sox21* in the lens this would result in weaker evolutionary constraints acting on the *Sox21* lens enhancers. As a consequence, to prevent their loss from the genome a second mechanism as for example overlap of enhancer functions would be needed to guarantee Sox21 lens expression and robustness of lens development. Therefore, CNEs may represent clusters of at least partially overlapping enhancers whose function is both to regulate developmental genes and to guarantee the robustness of the developmental programme.

## Materials and methods

### Identification of zebrafish CNEs and transgenic assays

Table S1 shows how the 19 CNEs tested in this study correspond to the CONDOR database entries for *Sox21* (http://condor.nimr.mrc.ac.uk/). The zebrafish CNEs were identified by using the BLASTN tool at the ENSEMBL genome browser (http://www.ensembl.org/Multi/blastview). We used the corresponding Fugu sequences as a query and BLAST settings of medium sensitivity. To confirm the absence of CNE 6 and CNE 17 from the zebrafish *sox21a* locus we used BLASTN again but with the zebrafish sequences identified at the *sox21b* locus as a query. The absence of hits at the *sox21a* locus confirms the absence of highly conserved lens enhancers but does not rule out the presence of regulatory sequences orthologous to CNE 6 and CNE 17.

The co-injection assay was performed as previously (Muller et al., 1997; Woolfe et al., 2005). The screening was performed approximately at 30 hpf and 52 hpf. To test CNEs in the tol2 system (Kawakami et al., 2004) we used the same expression vector and cloning strategy as described in (Fisher et al., 2006). The only modification was that to generate an entry clone for the Gateway technology (Invitrogen) CNEs were amplified by PCR and cloned into the pCR8/GW/TOPO vector (Invitrogen). Primer sequences for the co-injection assay and the generation of tol2-constructs are listed in Table S4. Lens expression of CNE 6 and CNE 17 was confirmed in three (CNE 6) or five (CNE 17) independent transgenic lines each.

### In situ hybridisations

In situ hybridisations were performed essentially as described in (Thisse and Thisse, 1998). To generate a *GFP* template the *GFP* coding region was amplified from the tol2-*GFP* vector (Fisher et al., 2006) using a reverse primer containing a T7 RNA polymerase binding site. Since both zebrafish *Sox21* genes consist of just one exon templates for *sox21a* and *sox21b* were generated directly from genomic DNA also by using reverse primers carrying a T7 RNA polymerase promoter. Primer sequences are listed in Table S5.

### BAC recombineering and injection

A Fugu BAC (accession number: BAC278I13) containing the entire *Sox21* locus with all CNEs was identified from a Fugu BAC library (available on request). The genomic sequence included in the BAC corresponds to Fugu scaffold_103:92028–154081 (www.ensembl.org) or chrUn:175972323–176034376 (genome.ucsc.edu) (Fig. 3A). Fig. 3A was retrieved from the UCSC genome browser and manually annotated. We substituted the single *Sox21* exon with the *EGFP* coding region (Clontech) by BAC recombineering (Lee et al., 2001). Recombineering was performed in *E. coli* strain EL350 (Lee et al., 2001) using an *EGFP*-Kanamycin cassette (Lakowski et al., 2007) to replace the *Sox21* exon. The targeting construct was amplified from a plasmid using the following primers:

Forward: TCGTACTTACTCTTATTCTATAATTATATTTCAGAAACTTGTGTGCCAACGTGAGCAAGGGCGAGGAG
Reverse: CTTTTTTTCTTGTGTTGATTTCACAATTTGGATAAAAGTCCAAAGCGGGCTATTCCAGAAGTAGTGAG

Bases corresponding to vector sequences are underlined. The remaining oligo sequence corresponds to genomic sequences flanking the Fugu *Sox21* coding region. BAC and PCR product were purified for electroporation using the ‘illustra GFX PCR and Gel Band Purification Kit’ (GE Healthcare).

We generated a second targeting vector to achieve the removal of multiple CNEs from the *Sox21:GFP* BAC. Our strategy was to replace CNEs by a selectable marker that could be removed again using Cre but leaving behind a non-functional *lox* site that would not interfere with additional rounds of Cre-driven homologous recombination. The targeting vector contains a Kanamycin resistance gene flanked by two modified *loxP* sites. Both *lox* sites contain a mutated central spacer region (Lee and Saito, 1998) to prevent interaction with the WT *loxP* site in the backbone of the BAC vector *pBeloBAC 11*. Secondly, both *lox* sites carry mutations in the distal arm with respect to the Kanamycin resistance gene and only the proximal arms correspond to the WT *loxP* sequence (Albert et al., 1995). These semi-WT lox sites recombine to generate one WT *loxP* site, except for the spacer, that is lost from the BAC together with the resistance gene leaving behind a fully mutant site without any WT arm which therefore is not recognized anymore by the *Cre* recombinase. This allows for using the same targeting cassette again to delete a second region from the same BAC. The sequences of the *lox* sites are:

upstream *lox* (*lox71 5171*): TACCGTTCGTATAGTACACATTATACGAAGTTAT
downstream *lox* (*lox66 5171*): ATAACTTCGTATAGTACACATTATACGAACGGTA

The numbers given to the modified lox sites are according to Lee and Saito (1998) and Albert et al. (1995). To remove the CNEs the *Sox21:GFP* BAC was transformed into the EL250 strain carrying an arabinose-inducible *Cre* recombinase gene (Lee et al., 2001). The Kanamycin-lox cassette was amplified by PCR and introduced into the same cells containing the BAC. Successfully modified BAC clones were isolated by screening for Kanamycin resistance. In order to remove the selectable marker cells were induced in 0.1% arabinose/LB medium for 1 h and then screened for Kanamycin sensitivity. To remove a second CNE from the same BAC the procedure was repeated just using different primers to amplify the Kanamycin-lox targeting cassette. Primer sequences to generate the deletions given in Table S6.

All BAC clones were purified using the ‘NucleoBond BAC 100’ kit (Macherey-Nagel) and we injected 30 ng/μl supercoiled BAC DNA in 0.5% phenol red and salts (5 mM Tris pH8, 0.5 mM EDTA, 1 mM KCl) (Meng et al., 1997). Embryos were screened at around 52 hpf under an Olympus IX81 fluorescence microscope. To measure lens expression we first discarded all embryos with less than 100 *GFP*-positive cells in the spinal cord. In the remaining embryos eyes were imaged under constant camera settings and intensity measurements were conducted using analysis tools included in the ImageJ software (Abramoff et al., 2004). Average intensities correspond to the total intensity divided by the size of the lens and maximum intensities count lenses in which the brightest pixel reaches the maximum intensity value of 255.

### Morpholino injections and morpholino rescue

The Sox21b morpholino directed against the 5'UTR (CCTGCTTCAGGTAGAAATCCACTGA) as well as a 5-base mismatch control morpholino (CCaGgTTCAcGTAcAAATCCACTcA) (bases that differ from the Sox21b morpholino are printed in lower case and underlined) were obtained from Gene Tools. We injected 4.25 ng per embryo of both morpholinos diluted in Danieau buffer (Nasevicius and Ekker, 2000) into one-cell stage zebrafish embryos.

To rescue the morpholino-induced phenotype we generated a *sox21b* mRNA carrying 5 base pair changes in the 5'UTR corresponding exactly to the mutations included in the mismatch morpholino. Due to inconsistencies observed in the number of rescued morphants we increased mRNA stability by substituting the WT 3'UTR by an SV40 poly A signal. The single WT *sox21b* exon was amplified from genomic DNA using the following primers

Forward-EcoRI: TATGAATTCCCAGGATTTACCAAGGATG
Reverse-HindIII-A: CATAAGCTTTGACAGCATTGACCGATCTT

The PCR product was then cloned into pBluescript (Stratagene). Mutations were introduced into the 5'UTR by amplifying this region in a second PCR using the same forward primer as before together with TGATCCATGGGCTTGGACATGACGCGTTCACCAGGTTCACGTACAAATCCACTCACA. Bases that do not correspond to the WT sequence are underlined. The WT 5'UTR was exchanged in the *pBluescript* clone by using *EcoRI* together with a *NcoI* site in the *sox21b* sequence.

The SV40 polyA signal was added by amplifying the *sox21b* mRNA including the mutated 5'UTR but not the WT 3'UTR with the following primers:

Forward = Forward-EcoRI (see above)
Reverse-HindIII-B: ATAAAGCTTAAACTTTTCTCGCTTTAGAGTCTCAT

The PCR-product was cloned into pBluescript (Stratagene). The SV40-polyA sequence was derived from the tol2-*GFP* vector (Fisher et al., 2006) and amplified with this primer pair:

SV40-Forward-HindIII: ATAAAGCTTGGATCATAATCAGCCATACCA
SV40-Reverse-XhoI: ATACTCGAGAAGATACATTGATGAGTTTGGACA

This PCR-produce was then cloned behind the previously amplified sequence containing the mutated 5'UTR and the *sox21b* coding region.

A template to synthesize mRNA for injections was amplified with a forward primer including a SP6 RNA polymerase promoter (TACGATTTAGGTGACACTATAGAACCAGGATTTACCAAGGATGC) (SP6 sequence is underlined) and the SV40-Reverse-XhoI primer. The mRNA was synthesized using the ‘mMESSAGE mMACHINE’ kit (Ambion) and purified using ‘SigmaSpin’ Sequencing Reaction Clean-UP columns (Sigma).

For the rescue experiment we added 100 ng/μl of a modified *sox21b* mRNA to the morpholino injection mix.

### Dissection and mutagenesis of CNE 6

We first used JASPAR (http://jaspar.cgb.ki.se/) (Sandelin et al., 2004a) to scan the sequence of Fugu CNE 6 for putative Sox binding sites. There are different binding matrixes for Sox factors included in the JASPAR database but all include a recognition sequence similar to ^C^/_T_^T^/_A_TTG^T^/_A_. This sequence is best represented by the Sox17 and Sox10 position weight matrixes (PWMs) and we used these matrixes and a threshold of 85% for the scan. We did not use the Sox2 PWM in JASPAR because it is probably a combination of a Sox2 site and a second binding site (possibly for Oct4) (Chen et al., 2008; Loh et al., 2006) because only the first half corresponds to the motif given above. Next we generated an alignment using ClustalW2 (http://www.ebi.ac.uk/Tools/msa/clustalw2/) using the Fugu CNE 6 sequence and othologous sequences from other vertebrates. The human and mouse sequences were retrieved directly from CONDOR (http://condor.nimr.mrc.ac.uk/index.html). The zebrafish sequence was retrieved as described earlier. To identify the chick and frog sequences we used BLASTN at the ENSEMBL genome browser (http://www.ensembl.org/Multi/blastview) and both Fugu and Human CNE 6 sequences as query. BLASTN sensitivity was set to medium and the longer hit of both query sequences was used for the alignment. We then tried to identify Sox sites at roughly corresponding positions in the other vertebrate sequences. A site was considered for further analysis if for Fugu JASPAR had returned a score > 0.9 and the site was conserved in at least one other species or if for Fugu JASPAR had returned a score between 0.85 and 0.9 and the Sox sites could be found in all other sequences.

Oligos used for the dissection and mutagenesis of CNE 6 are listed in Table S7. Since sites 1 and 6 are at the very 5' and 3' end of CNE 6 these mutations were introduced by amplifying CNE 6 using a modified forward or a modified reverse primer. Mutations in sites 2 to 4 were generated by mutating the WT sequence already inserted into the tol2 vector using the ‘QuickChange II Site-Directed Mutagenesis Kit’ (Agilent Technologies) (Sox sites 2, 3, 4, and 5). Embryos were screened under a Leica MZ 16F dissecting microscope for lens expression after discarding those that showed no or only very weak expression in the CNS at 52 hpf. From the remaining embryos we randomly picked at least 30 embryos (or 60 lenses) and counted the number of lenses positive for GFP. This was repeated between two and five times for each construct.

## Appendix A Supplementary materials

Fig. S1. Mutational analysis of putative Sox binding sites in CNE 6. (A) Sequence alignment of CNE 6 orthologues from 5 different vertebrates. Putative Sox sites considered in this study are highlighted in red. The central part of the Fugu sequence marked in grey corresponds to region 'B' in the deletion analysis. (B, C) Transient GFP-expression in 52 hpf old zebrafish injected with reporter plasmids in which GFP is controlled by the Fugu WT version of CNE 6 (B) or Fugu CNE 6 carrying a mutation in Sox site 4 (C). Note that in (C) absence of lens expression coincides with absence of GFP-expression in the telencephalon (arrows).
