# Supplementary material for: Lens development depends on a pair of highly conserved Sox21 regulatory elements
Source: Dev Biol. 2012 May 1;365-248(1):310–8. doi: 10.1016/j.ydbio.2012.02.025 (PMC3480646; doi:10.1016/j.ydbio.2012.02.025)

SUPLLEMENTARY TABLES

TABLE S1

CNE number and corresponding accession numbers in the CONDOR database (http://condor.nimr.mrc.ac.uk/)

CNE 1	CRCNE00000565
CNE 2	CRCNE00000566 + CRCNE00000567
CNE 3	CRCNE00000568 + CRCNE00000569 + CRCNE00000570
CNE 4	CRCNE00000571
CNE 5	CRCNE00000572
CNE 6	CRCNE00000573
CNE 7	CRCNE00000596 + CRCNE00000574
CNE 8	CRCNE00000597 + CRCNE00000575
CNE 9	CRCNE00000576 + CRCNE00000577
CNE 10	CRCNE00000578
CNE 11	CRCNE00000579
CNE 12	CRCNE00000580
CNE 13	CRCNE00000581
CNE 14	CRCNE00000582 + CRCNE00000583
CNE 15	CRCNE00000584 + CRCNE00000585 + CRCNE00000586 + CRCNE00000617
CNE 16	CRCNE00000587
CNE 17	CRCNE00000589 + CRCNE00000590
CNE 18	CRCNE00000591
CNE 19	CRCNE00000592 + CRCNE00000593

TABLE S2. Percentages of individuals with GFP expression at 30 hpf.

CNE	1	2	3	4	5*	6	7	8	9	10	11	12	13	14	15*	16	17	18	19
forebrain	0	3.92	6.25	18.92	0	78.18	13.20	75.00	43.48	100	0	16.67	78.95	3.23	0	23.08	72.22	0	17.07
midbrain	17.07	0	6.25	13.51	0	7.27	1.90	79.17	4.35	80.56	3.23	0	10.53	0	0	0	50.00	0	14.63
hindbrain	14.63	13.73	6.25	43.24	0	16.36	5.70	87.50	4.35	97.22	3.23	11.11	0.00	0.00	0	7.69	61.11	0	26.83
sp. cord	0.00	3.92	0	51.35	0	12.73	7.50	54.17	0	75.00	0.00	44.44	10.53	6.45	0	0.00	69.44	0	0
retina	0	1.96	0	0	0	33.33	0	70.83	8.7	69.44	0	0	0	0	0	0	52.78	0	0
lens	0	0	0	0	33.33	29.63	1.89	16.67	4.35	0	0	0	0	0	0	0	13.89	0	0
ear	0	9.80	0	0	0	1.81	75.50	12.50	0	72.22	0	33.33	0	35.48	100	26.92	0	0	0
notochord	0	62.75	0	0	0	1.81	0	8.33	0	0	0	5.56	0	0	0	3.85	8.33	0	0
muscle	0	0	0	2.70	66.66	0	0	12.50	4.35	30.56	0	11.11	0	3.23	0	3.85	8.33	0	36.59
blood	0	3.92	6.25	2.70	0	0	0	4.17	0	2.78	3.23	5.56	15.79	6.45	0	11.54	0	0	34.15
heart	0	0	0	2.70	00	0	0	0	0	2.78	6.45	0.56	15.79	58.06	0	0	2.78	0	0
skin	53.66	11.76	62.50	16.22	0	5.45	20.80	16.67	43.48	66.67	29.03	22.22	0	9.68	0	34.62	16.67	0	17.07
fins	34.15	3.92	50.00	5.41	0	0	1.90	12.50	0	8.33	12.90	5.56	5.26	0	0	0	0	0	4.88
other	31.71	11.76	18.75	8.12	0	9.09	13.21	12.50	17.39	25.00	74.19	22.22	31.58	3.23	0	3.85	22.22	0	26.83
embryos	189	364	49	130	81	200	370	28	71	40	252	44	54	212	125	270	46	117	92
%positives	21.69	14.01	32.65	28.46	3.70	27.50	14.32	85.71	32.39	90.00	12.30	40.91	35.19	14.62	0.80	9.63	78.26	0	44.57

'Embryos' shows the total number of injected embryos for each single CNE and '%positives' the percentage of positive embryos in total for each single CNE. The numbers for different anatomical domains correspond to the percentage of positive embryos with GFP expression in that area.

* Note that for these CNEs the percentage of positive embryos in certain anatomical domains is high because the total number of positive embryos is very low. We do not consider these CNEs to show any enhancer activity in this assay.

TABLE S3. Percentages of individuals with GFP expression at 52 hpf.

CNE	1	2	3	4	5*	6	7	8	9	10	11	12	13	14	15*	16	17	18	19
forebrain	0	8.70	13.33	25.53	0	35.29	13.40	62.50	29.63	85.71	13.33	16.00	70.59	2.94	0	13.04	30.56	0	29.73
midbrain	31.82	4.35	33.33	23.40	0	23.53	3.00	50.00	7.41	85.71	20.00	12.00	47.06	0	0	10.87	47.22	0	64.86
hindbrain	4.55	17.39	26.67	65.96	0	47.06	16.40	81.25	37.04	96.43	33.33	4.00	23.53	11.76	100	15.22	72.22	0	13.51
sp. cord	0	0	6.67	48.93	0	35.29	3.00	62.50	3.70	75.00	20.00	8.00	0	2.94	0	2.17	66.00	0	0
retina	0	0	0	0	0	11.76	0	59.38	3.7	33.33	0	4	5.88	0	0	0	2.78	0	0
lens	4.55	0	0	0	0	47.06	2.99	3.13	3.7	0	0	0	1	0	0	0	33.33	0	0
ear	0	8.70	0	0	0	0	76.10	0	3.70	53.57	0	36.00	5.88	44.12	0	43.48	11.11	0	0
notochord	0	56.52	0	0	0	0	0	3.13	0	0	0	4.00	0	0	0	2.17	0	0	0
muscle	0	0	0	0	100	0	0	3.13	0	10.71	0	12.00	0	2.94	0	4.35	0	0	10.81
blood	0	0	0	0	0	0	0	0	0	3.57	3.33	0	0	0	0	0	0	0	5.41
heart	0	0	0	2.13	0	0	0	6.25	0	0	13.33	0	5.88	61.76	0	2.17	0	0	0
skin	45.45	13.04	20.00	4.26	0	0	0	6.25	40.74	10.71	13.33	28.00	0	14.71	0	23.91	0	0	2.70
fins	36.36	4.35	26.67	0	0	0	0	3.13	3.70	0	3.33	12.00	5.88	0	0	0	0	0	0
other	18.18	13.04	13.33	4.26	0	5.88	10.45	0	14.81	3.57	36.67	48.00	5.88	5.88	0	0	5.56	0	13.51
embryos	154	182	41	109	74	44	206	38	64	29	179	36	30	212	98	263	44	105	81
%positives	14.29	12.64	36.59	43.12	1.36	38.64	32.52	84.21	42.12	96.56	16.76	69.44	56.67	16.04	1.02	17.49	81.82	0	45.68

'Embryos' shows the total number of injected embryos for each single CNE and '%positives' the percentage of positive embryos in total for each single CNE. The numbers for different anatomical domains or tissues correspond to the percentage of positive embryos with GFP expression in that area.

* Note that for these CNEs the percentage of positive embryos in certain anatomical domains is high because the total number of positive embryos is very low. We do not consider these CNEs to show any enhancer activity in this assay.

TABLE S4

Primer sequences used to amplify Fugu (*Tr*) and zebrafish (*Dr*) CNEs

CNE	forward primer	reverse primer
**Tr1**	ATTGCATTGTAAAGTGCCTATTT	CCCCATAGAGAGAGATAAACCA
**Tr2**	TTATTTCCAAAACGGGGTTG	AAAGCCATCCTCTGTCATCG
**Tr3**	TTCCTGTAAAATTCATTTGTGATG	AAAAGGAGCGGAGGGTGATA
**Tr4**	CTGAAAGACCCCATCAGACC	ATAGGGTTTCCAGGGCTGTC
**Tr5**	CCTTGAGAGGCCACCAAAT	GCCCTCCACTTTTCTTTTTC
**Tr6**	CTGGGCCATGAGAGCGG	CAGGCATGCATCTGAAGC
**Tr7**	CCACGGTGACCTTCATGC	CTGCCAGCTCCTTCTGAG
**Tr8**	GATGAGGCTGAAACACAAAGG	GCCTCAGAGGGGGATGAAT
**Tr9**	AAGTAGATAAATGCCCTCCAAG	ATCTCACGCATCTGCACTC
**Tr10**	GCTTTTCCCACCCCTAAACT	CCATAAGTCATACAAGCTGC
**Tr11**	GTCAGACAGGGGGTGTGATT	GCAGCGGTATCAAATTGCTT
**Tr12**	GCTGAAAAGAATACAGGCAATG	AACATTGAGTCATACTCAGGC
**Tr13**	CTGACCAAGCTTCAGCTTC	GTGCACTGCAACTGCAAGA
**Tr14**	CAACTGGAACTATAAATGTATC	ATTCGAGGCTGCACGGTGC
**Tr15**	TCTTCTGGAATTTGTATGTTTTGTG	AGGCCGGTCCTGGTTCTA
**Tr16**	CACGTGGACGTGAGAGTG	TCCTGACAGATCTGCGCG
**Tr17**	GGAAGAAAGCTGAAGGAAC	CCACCTTTTGCATTCACTC
**Tr18**	GTTTCCAAGGGAGGGGAAA	CTGTGTCCCGTGTTGGAG
**Tr19**	GACACCCGGTTCCATTCTT	CGTCAAACTTTCCAGAGCG
**Dr6**	AGGGGTTGTTTGGGTCTTTT	CCACACAGACTGGAATAGTGTAGC
**Dr12**	TAAACAGTGGCGAAGAATGG	CCTCCGCAAGCTGTACCTTC
**Dr17**	GGGGCACTAACTGAAAGAA	CGTTTCCAGAAAAGCACAGA

TABLE S5

Oligos used to generate templates for RNA probes. The T7 promoter sequence is underlined.

GFP:
Forward: CAAGGGCGAGGAGCTGTT
Reverse: TAATACGACTCACTATAGGGCTTGTACAGCTCGTCCATGC

Zebrafish *sox21a*:
Forward: AGCATTTAAAGCCAGGTCAC
Reverse: TAATACGACTCACTATAGGAATGGTTAGCATGTAAACAAGAGC

Zebrafish *sox21b*:
Forward: AGCGTGAAGTAAACCTGGAGA
Reverse: TAATACGACTCACTATAGGAGACTGGATCCGTTCAGCAC

TABLE S6

Primers used to amplify the targeting constructs for the removal of CNEs from the BAC. Vector sequences are underlined.

**CNE 6 forward:**	TTCCATTTGGCCGGGGGGGCTTTTATACTGGGCCATGAGAGCGG<br>TACCGTTCGTATAGTACACATTATACGAAGTTATGC
**CNE 6 reverse:**	AGGCATGCATCTGAAGCAACATAGGAAAGATAATGTATCCCAAG<br>TACCGTTCGTATAGTACACATTATACGAAGTTATCT
**CNE 8 forward:**	TTCAGTGGCGGGTGATGAAAGAGATGAGGCTGAAACACAAAGGC<br>TACCGTTCGTATAGTACACATTATACGAAGTTATGC
**CNE 8 reverse:**	CCTACTTTAATTTCCCTGCAGGCCTCAGAGGGGGATGAATTCAA<br>TACCGTTCGTATAATGTGTACTATACGAAGTTATCT
**CNE 17 forward:**	TGGGAAGAAAGCTGAAGGAACTTTTTTTTCCCCCCTCCAAACTC<br>TACCGTTCGTATAGTACACATTATACGAAGTTATGC
**CNE 17 reverse:**	CCACCTTTTGCATTCACTCCCCATCTCAGACAGAAAAAACACAG<br>TACCGTTCGTATAATGTGTACTATACGAAGTTATCT

TABLE S7

Oligo sequences for the dissection of Fugu (*Tr*) CNE 6. Bases different from the WT sequence are highlighted in yellow.

construct	forward	reverse
Tr6-ABC	TGTCTGCTCGCATTTACGTG	AATGTATCCCAAGGCTACAAAA
Tr6-AB	TGTCTGCTCGCATTTACGTG	TTTGAGAGAGCTGACAGTAACCA
Tr6-BC	GGCTTCCTGTTCATTAGACTTCTT	AATGTATCCCAAGGCTACAAAA
Tr6-A	TGTCTGCTCGCATTTACGTG	GGCTTGCATAAACACTATTTAAAAC
Tr6-B	GGCTTCCTGTTCATTAGACTTCTT	TTTGAGAGAGCTGACAGTAACCA
Tr6-C	AAGCCATTGTCGTCCATTGT	AATGTATCCCAAGGCTACAAAA
Tr6-soxm1	GGCCCACTGCATCTTTTCGG<br>GTTACTTTCTCATCA	AATGTATCCCAAGGCTACAAAA
Tr6-soxm2	GGTGGCAAGCGCATGATGCG<br>CCTGTTGTATG	CATACAACAGGCGCATCATGCG<br>CTTGCCACC
Tr6-soxm3	TCTTTTCACCTCTGCATGATG<br>CGCCGGCTTTG	CAAAGCCGGCGCATCATGCAGA<br>GGTGAAAAGA
Tr6-soxm4	GTGTTTATGCAAGCCATCATC<br>GTCCATTGTGCGTC	GACGCACAATGGACGATGATGG<br>CTTGCATAAACAC
Tr6-soxm5	CCATTGTCGTCCATCATGCGT<br>CCCGTCCTAATG	CATTAGGACGGGACGCATGATG<br>GACGACAATGG
Tr6-soxm6	TGTCTGCTCGCATTTACGTG	CCAAGGCTATGAAAGCAGGCAA<br>ATCACTGTGC

SUPPLEMENTARY FIGURE S1

A

```
human           AAGTGCCCACAAAGA-GGATTCCTTATGCTTTAATTTCTCTTTATTTTTCTCACCGTAAA 59
chicken         AAGTGCCTACAAAAAAGGATTCCTTATGCTTTAATTTCTCTTTATTTTTTTGACTGTAAA 60
mouse           AAATGCCCACAAAGA-AGACTCCTTATGCTTTAATTTCTCTTTATTTT-CCGACTGTCAA 58
frog            ----------------GGAATCCATGT-TTTCAGCTATTCTTTATTTCAGGGACTGTAAA 22
zfish           ---------------------------GGGCGACTTTCTCATCACTCCTCCTAATGCTG- 32
fugu            ------AAGGGCCCACTGTGTCTTTTCGGGTTACTTTCTCATCATTTCTCCTAATGTAGA 54
                                                *  *  ** * * *      *  *

human           GACCCTTGGGGTAAATGCCAGCCCCACAATCCCGCCGTTATATGGTTACTGTCAGCCTGC 119
chicken         GACCCATGGGGTAAATGCCAGTCCCACAATCCTGCAGTTATATGGTTACTGTCAGCCTGC 120
mouse           GACCCTCGGGGTAAATGGCAG-CCCACAATTCCATCTTTAGATGGTCACTGTCAGCCTGC 117
frog            GGCCTATGGGGTAAATGCCAGTCCCTTAATCCTGCAGTTATATGGTTACTGTCAGCCCGT 82
zfish           GACCCACAGGGTCGATGGCAGCCCCACAATGCATCTGTTGTATGGTTCCTGTCAG----- 87
fugu            GACCCACAGGGTTGGTGGCAAGCGCACAATGCGCCTGTTGTATGGTTACTGTCAGCTCTC 114
                * **    ****   ** **  * *  *** *     **  *****  *******

human           TCAAATGATGCTTCCTGTTCATTAGGCCTCTTTTCATCTGACTACAATGTCTTGGCGTTG 179
chicken         TCAAATGATGCTTCCTGTTCATTAGGCGTCCTTTCATCTGACAACAATGTACTGGCTTTG 180
mouse           TCATATGATGCTTCCTGTTCATTAGGTCTCTTTTCATCTGACTACAATGTCCCGGCTTTG 177
frog            TCAAATGATGCTTCCTGTTCATTAGACCTCCTTTCATCTAACAACAATACACCGGCTTTG 142
zfish           -----TAAGGCTTCCTGTTCATTAGGCATCTTTTCACCTCCGCACAATAAGCAGATTCAG 142
fugu            TCAAATAAGGCTTCCTGTTCATTAGACTTCTTTTCACCTCTGCACAATGCGCCGGCTTTG 174
                     * * ****************   ** ***** **    *****     *     *

human           CAGCAATTCTTAGTCAACGCCCATCTCTTCAATAGCCTGTGAATAGTATTAAATGGTGTT 239
chicken         CTGCAATTCTTAGTCAACGCCCATCTCTTCAATAGCCTGTGAATAGTATTAAATAGTGTT 240
mouse           CTGCAATTCTCCCTCAACGCCCATCTCTTCAATAGCCTGTGAATAGTATTAAATGGTGTT 237
frog            CTGCAATTCTTAGTCAACGCCCATCTCCTCAAAGGCTTGTGAATAGTATTAAATAGTGTT 202
zfish           CTGCAATGCTGCGTCGCCGCCCAGCCCCTCAATGCCGCGTCAAAGCCTCTAAATGCTGTT 202
fugu            CAGCAATAGTGAGTTGACGCCCATCTCTTCAATGCCATGTGAATAGTTTTAAATAGTGTT 234
                * *****  *   *   ****** * * ****   *  ** **      *****  ****

human           TATGCTAGCTATTGTTGCCTTTTGTGTGGTTTGTC-TAATTCACAGAGAAAAGTCTGCTT 298
chicken         TATGCTAGCTATTGTTGCCTTTTGTGTGGTTTGTC-TAATTCACAGAGAAAAGTCTGCTT 299
mouse           TATGCTAGCCATTGTTGCCTATTGTGTGGTTTGTC-TAATTCACAGAGAAAAGTCTGCCT 296
frog            TATGCTACCTATTGTTGCCCTTTGTGTGGTATGTC-TAATTCACAAAGAAAGGTCTG-CT 261
zfish           TATGCACGCCATTGTTGCTCTTTGTGCGGCCGCTTTTAATGCGCAGCGATTCGTCTGCTT 262
fugu            TATGCAAGCCATTGTCGTCCATTGTGCGTCCCGTCCTAATGCACAGTGATTTGCCTGCTT 294
                *****   * ***** *    ***** *     *  **** * **  **   * ***  *

human           TTGT--- 302
chicken         TTGT--- 303
mouse           TTGT--- 300
frog            TTGTG-- 262
zfish           TTGTGGC 269
fugu            TTGTAGC 301
                ****
```

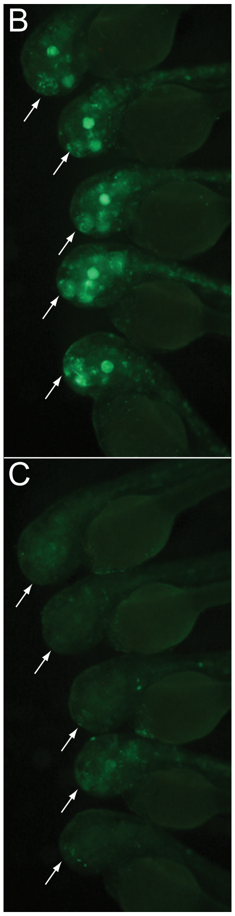

Supplement: Supplementary file 1 — Fig. S1. Mutational analysis of putative Sox binding sites in CNE 6. (A) Sequence alignment of CNE 6 orthologues from 5 different vertebrates. Putative Sox sites considered in this study are highlighted in red. The central part of the Fugu sequence marked in grey corresponds to region 'B' in the deletion analysis. (B, C) Transient GFP-expression in 52 hpf old zebrafish injected with reporter plasmids in which GFP is controlled by the Fugu WT version of CNE 6 (B) or Fugu CNE 6 carrying a mutation in Sox site 4 (C). Note that in (C) absence of lens expression coincides with absence of GFP-expression in the telencephalon (arrows). [file mmc1.pdf]
